# Prof. James B. Hodgkin, D. D. S.

**Published:** 1877-10

**Authors:** 


### EDITORIAL, ETC.

---

*Prof. James B. Hodgkin, D. D. S.*—It affords us pleasure to announce, that James B. Hodgkin, D. D. S., Professor of Dental Mechanism and Metallurgy, in the Baltimore College of Dental Surgery, has connected himself with this Journal in the position of Assistant Editor. Prof. H. is not unknown to our readers as he has frequently contributed articles which have proved interesting and instructive. We will, however, allow him to speak for himself.—*Senior Editor.*

---

The Associate makes his bow. Taking place as a junior in the ranks of journalism, he naturally feels diffident and sensitive of criticism. Time may dull both these qualities. He comes to the post with a good share of earnestness, which it is hoped will compensate for any crudeness; and trusts that as perseverance is essential to success, he may not lack this also. Earnestness and perseverance, then, on his part, and the kind indulgence of friends and readers,—with these and health, perhaps a little may be done for our profession, ennobled by many choice spirits, and degraded, sadly enough, by other spirits which grovel.

The position of editor of a dental journal should not be different from that occupied by those in charge of other literary work of like character, yet it seems to be. The editor should feel perfectly free to criticise what is expressed by writers and speakers in the profession. Yet, by a singular condition of affairs, what is allowed in other journalism seems to be fought shy of by those conducting the dental journals. Why, it is hard to say, unless the profession is not sufficiently advanced for such reviewing.

Cannot our writers and speakers take a step forward with us, and accept honest criticism in the spirit of truth and sincerity? As it is there seems to be offence in the idea of this,—an impression that to be critical, means to be personally hostile. We are sorry to write these words, but no one can attend a dental convention, or read consecutive numbers of a dental journal without feeling that this is true. It ought not to be.

By this time the idea dawns on the mind of the reader that the Associate means to set himself up as a reviewer, and unmercifully turn iconoclast. By no means; he has neither taste or time for such work. What he wishes to say is that if the judgment of the editors should dictate the elimination of a crudity in an article sent for publication, or should briefly comment on, or dissent from the views expressed by the writers of original communications,—and we hope to see these coming thick and fast,—that the writers will not feel sufficiently sensitive to take such criticism as a personal matter. This is what the Associate has to say on this part of his subject.

He has to say also that the editors are never so delighted as when they receive sprightly, clearly written articles for publication; and we would like to truthfully say that all that come are of this class, but cannot conscientiously do so. Friends who write, try and have clearly before you your subject matter in detail, as well as the subject. To do this will involve labor, with brains and pen; but better that *you* should work a little or a great deal, to make your meaning clear, than that your reader should labor in vain to understand what the writer intended but failed clearly to express. Unless you are a professional writer it is hazardous to send out your written thoughts without a reviewing, perhaps more than once, and the best composers in the higher walks of literary life go over and over again their manuscript. Take your time about this: your article is not to be patented, and you are in slight danger of being forestalled in the publication of your discoveries. The common expression: "I write hurriedly," is not always perfect candor. Take your time, be sure you have something to say, and say it your very best.

Another word: The editors have pretty good eyes, but they are obliged, such is the poor patronage of dental journals, to work at their chosen calling of dentistry, and are not anxious that the sight which is already severely taxed in operating shall be further strained by illegible copy. Often the difficulty of deciphering manuscript is so great as to cause the reader to pass over some words, or blindly guess at their meaning, in the hope that the printer will make out what the editor cannot. And the printer, though expected to be able to read all things, is but a mortal, and sometimes a profane one. So then, for the sake of the editors' eyes, and the printers' temper, write as plainly and legibly as possible.—These matters do not have special bearing on dentistry, but they do on journalism, and hence we write of them. J. B. H.

